# Predicting 72-hour and 9-day return to the emergency department using machine learning

**DOI:** 10.1093/jamiaopen/ooz019

**Published:** 2019-07-01

**Authors:** Woo Suk Hong, Adrian Daniel Haimovich, Richard Andrew Taylor

**Affiliations:** 1 Yale School of Medicine, New Haven, Connecticut, USA; 2 Department of Emergency Medicine, Yale School of Medicine, New Haven, Connecticut, USA

**Keywords:** decision support techniques, emergency medicine, machine learning

## Abstract

**Objectives:**

To predict 72-h and 9-day emergency department (ED) return by using gradient boosting on an expansive set of clinical variables from the electronic health record.

**Methods:**

This retrospective study included all adult discharges from a level 1 trauma center ED and a community hospital ED covering the period of March 2013 to July 2017. A total of 1500 variables were extracted for each visit, and samples split randomly into training, validation, and test sets (80%, 10%, and 10%). Gradient boosting models were fit on 3 selections of the data: administrative data (demographics, prior hospital usage, and comorbidity categories), data available at triage, and the full set of data available at discharge. A logistic regression (LR) model built on administrative data was used for baseline comparison. Finally, the top 20 most informative variables identified from the full gradient boosting models were used to build a reduced model for each outcome.

**Results:**

A total of 330 631 discharges were available for analysis, with 29 058 discharges (8.8%) resulting in 72-h return and 52 748 discharges (16.0%) resulting in 9-day return to either ED. LR models using administrative data yielded test AUCs of 0.69 (95% confidence interval [CI] 0.68–0.70) and 0.71(95% CI 0.70–0.72), while gradient boosting models using administrative data yielded test AUCs of 0.73 (95% CI 0.72–0.74) and 0.74 (95% CI 0.73–0.74) for 72-h and 9-day return, respectively. Gradient boosting models using variables available at triage yielded test AUCs of 0.75 (95% CI 0.74–0.76) and 0.75 (95% CI 0.74–0.75), while those using the full set of variables yielded test AUCs of 0.76 (95% CI 0.75–0.77) and 0.75 (95% CI 0.75–0.76). Reduced models using the top 20 variables yielded test AUCs of 0.73 (95% CI 0.71–0.74) and 0.73 (95% CI 0.72–0.74).

**Discussion and Conclusion:**

Gradient boosting models leveraging clinical data are superior to LR models built on administrative data at predicting 72-h and 9-day returns.

## INTRODUCTION

Emergency department (ED) returns represent an important quality of care metric and patient-centered outcome.^1^^,^^2^ Being able to predict a patient’s likelihood of returning to the ED may allow providers to engage in an evidence-based discussion regarding a patient’s discharge plan, provide optimal care for those who would have been prematurely discharged, and reduce ED overcrowding.^3^^,^^4^ While periods ranging up to 30 days have been used as a time frame for ED return, a shorter time frame may be useful in identifying return visits related to the previous episode of care as well as in identifying preventable causes of ED return.^5–8^ Many prior studies define early ED return as those occurring within 72 h of discharge,^1^^,^^9–14^ while, more recently, a time frame of 9 days has been proposed as an objective threshold for early return.^15^^,^^16^

Risk factors for ED return have been studied extensively.^10^^,^^17–21^ Prior studies have relied on a handful of variables used in traditional descriptive methods such as demographic variables and qualitative metrics (ie, disability rating and living situation).^17^^,^^18^ Recent studies have explored the utility of quantitative variables such as medication counts, diagnoses codes, and temporal markers of ED utilization.^10^^,^^11^^,^^19^^,^^22^^,^^23^ Machine learning algorithms such as random forests and gradient boosting have been used to predict ED return using the 30-day time frame,^19^^,^^23–27^ a cutoff likely borrowed from Medicare’s Hospital Readmission Reduction Program.^28^^,^^29^ However, study of shorter ED return intervals has been limited by the use of routine administrative data, small sample size, and use of linear algorithms such as logistic regression (LR).^11^^,^^12^^,^^15^ One such investigation of 72-h returns was restricted to a predominantly male, elderly Veteran population.^12^ While administrative data have been shown to contain many useful features, such as prior hospital utilization history and comorbidity categories, the predictive value of detailed clinical data such as lab values and ED administered medications has yet to be explored. Given their utility in predicting various patient outcomes, clinical data extracted from the electronic health record (EHR) may improve prediction of early ED return.^30–32^ Moreover, we hypothesize that the relationship between previously studied risk factors is nonlinear and may be more accurately modeled by nonlinear algorithms.^33^^,^^34^

Expanding on prior work, we predict both 72-h and 9-day ED return using a large dataset of adult ED visits, with 1500 variables extracted per visit from the EHR, including but not limited to: historical and current vitals and lab values, ECG and imaging counts, ED administered medications, and discharge diagnosis. To test the impact of additional input features, we build our models on 3 subsets of variables: administrative data (demographics, prior hospital usage, and comorbidity categories), data available at the time of triage, and data available at the time of discharge. We use gradient boosting (XGBoost), a powerful classification algorithm suited for EHR data due to its ease with missing values, to model nonlinear relationships.^27^^,^^30^^,^^31^^,^^35^^,^^36^ Finally, we discuss how such a prediction model can be used either to trigger intervention for patients with high return risk or to retrospectively identify lapses in care for quality assurance process.

## METHODS

### Study design and setting

Retrospective data on all adult visits was obtained from 2 EDs covering the period of March 2013 to July 2017 to ensure a 1-year historical time frame from the study start period of March 2014 as well as a 9-day prospective time frame for all visits. The represented EDs include a level 1 trauma center with an annual census of approximately 85 000 patients and a community hospital-based department with an annual census of approximately 75 000 patients. Both EDs are located within the same city and are part of a single hospital system utilizing the Epic EHR (Verona, WI, USA) and the Emergency Severity Index for triage. This study adhered to the Transparent Reporting of a multivariable prediction model for individual prognosis or diagnosis statement.^37^ This study was approved, and the informed consent process waived, by the Human Investigation Committee at the authors’ institution (IRB 2000022883).

### Response variable

Two binary outcomes were defined for every ED visit ending in discharge: return within 72 h and return within 9 days. Given the overlap in patient population and frequent transfers between the 2 EDs included in the study, a return visit at either ED was considered valid regardless of the location of the initial visit.

### Feature processing

The dataset used for this study derives from our prior study on predicting hospital admission at triage, which made use of patient demographics, time and location of presentation, triage vitals, chief complaint, hospital usage statistics, past medical history, outpatient medications, historical vitals, historical labs, and historical imaging and EKG counts.^30^ To these variables, we added variables collected during the patient’s current ED visit, including discharge diagnosis, ED administered medications, current lab values, current vitals, procedures and imaging orders, as well as the presence of a primary care provider listed on the EHR, for a total of 1500 variables. The chief complaint was encoded as a categorical variable with the top 200 most frequent values (>90% of all visits) as unique levels and all other values binned to ‘Other’. No natural language processing was used given that the dataset did not contain triage notes. The location of the encounter was encoded in the dataset (deidentified as “A” vs “B”), allowing the models to take into account differences in practice patterns between the 2 EDs. Processing steps for each variable category are outlined in Supplementary Text S1, while the full list of variables are provided in Supplementary Table S1. All data elements were obtained from the enterprise data warehouse, using SQL queries to extract relevant raw-data in comma-separated value format. All subsequent processing and analysis were done in R. The link to the repository containing all R scripts is available in Supplementary Text S1.

### Model fitting and evaluation

Samples were randomly split into a training set of 264 631 (80%), a validation set of 33 000 (10%), and a held-out test set of 33 000 (10%). All categorical variables were converted to numeric variables using one-hot encoding.^38^ For LR, all administrative variables were scaled to the interval between 0 and 1, then imputed using the median of each variable. No imputation was performed for XGBoost, since it learns a default direction for each split in the case that the variable needed for the split is missing.^35^ XGBoost models were trained for each of the 2 outcomes (72-h return, 9-day return) on 3 subsets of variables: administrative data (demographics, prior hospital usage, comorbidity categories), data available at the time of triage, and the full set of data available at the time of discharge (Table 1).

**Table 1.** Variables included by dataset type

Category	Number of variables	Administrative	Triage	Discharge
Response variable (72-h or 9-day return)	1	X	X	X
Demographics	10	X	X	X
Hospital usage statistics	4	X	X	X
Past medical history	281	X	X	X
Triage evaluation	13		X	X
Chief complaint	200		X	X
Outpatient medications	48		X	X
Historical vitals and labs	407		X	X
Prior imaging/ECG counts	9		X	X
Current vitals	19			X
Current labs and orders	135			X
ED administered meds	98			X
Discharge diagnosis	275			X
Total	1500	296	973	1500

*Abbreviations:* ECG: electrocardiogram; ED: emergency department.

LR models were trained on all samples excluding the test set using the RMSprop optimizer in the Keras interface.^39^ Hyperparameters for each XGBoost model were optimized by maximizing the AUC of the validation set. The optimized set of hyperparameters was then used to train the XGBoost model on all samples excluding the test set. The test AUC of each model was calculated on the held-out test set with 95% confidence intervals (CIs) constructed using DeLong’s method.^40^ The equality between every pair of AUC values for each outcome was also tested using DeLong’s method. Information gain, an importance metric that quantifies the improvement in accuracy of a tree-based algorithm from a split based on a given variable, was used to identify important variables for the full models, and a reduced model built from the top 20 variables for each outcome.^41^ To test the applicability of the model at the time of triage, statistical measures such as sensitivity and specificity were calculated for the model using data available at the time of triage, with 95% CIs constructed using 1000-fold bootstrap. Details of the model fitting process are provided in Supplementary Text S1.

## RESULTS

### Characteristics of study sample

**Figure 1.**
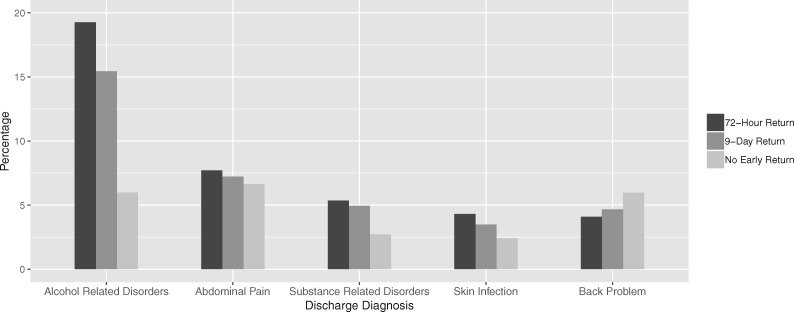
Frequent discharge diagnoses for visits that result in early ED return. The top 5 most frequent discharge diagnoses for visits that result in 72-h return are shown in order, as well as the respective percentages of those diagnoses for visits that result in 9-day return and for visits that do not result in early return. ED: emergency department.

From the 346 656 ED discharges in the study period, a total of 330 631 visits were available for analysis after filtering for inclusion criteria (age greater or equal to 18). Of these, 29 058 discharges (8.8%) resulted in a return visit within 72 h while 52 748 discharges (16.0%) resulted in a return visit within 9 days. Major characteristics of each outcome group are shown in Table 2. Patients who returned to the ED were more likely to be older, arrive by ambulance, have Medicaid, and have a higher number of prior ED visits. They were also more likely to have a history of chronic heart failure (CHF) or chronic obstructive pulmonary disease (COPD), as well as a history of alcohol or substance abuse. Alcohol-related disorders, abdominal pain, and substance-related disorders were the most frequent diagnoses for discharges resulting in early return (Figure 1). The risks of admission for 72-h and 9-day return visits were 22% and 20%, respectively, significantly lower than the general admission risk of 30% across all visits (*P* < .001).

**Table 2.** Characteristics of study sample

Variables	No acute return (*n* = 277 883)	72-h return (*n* = 29 058)	9-day return (*n* = 52 748)
Mean age (SD)	43.4 (18.1)	44.6 (16.5)	44.8 (16.9)
Gender (% male)	43	56	53
Arrival by ambulance (%)	27	44	41
Mean triage heart rate (SD)	84.5 (15.5)	87.2 (16.0)	86.7 (15.7)
Mean ESI level (SD)	3.24 (0.86)	3.02 (0.91)	3.06 (0.90)
Insurance status (% Medicaid)	41	53	53
Mean number of previous ED visits (SD)	2.44 (5.08)	17.2 (34.5)	13.8 (27.9)
Prevalence of COPD or CHF (%)	6	10	10
Prevalence of alcohol or substance abuse (%)	8	30	27

*Note:* All comparisons between visits that do not result in acute return and those resulting in early return  (either 72-h or 9-day return) were significant  (*P* < .001).

*Abbreviations:* CHF: chronic heart failure; COPD: chronic obstructive pulmonary disease; ED: emergency department; ESI: Emergency Severity Index; SD: standard deviation.

### Model performance

**Figure 2.**
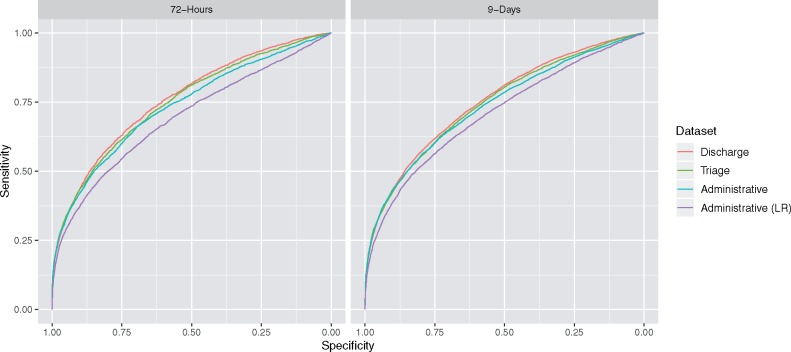
Receiver operating characteristic curves. The difference in AUC value for every pairwise comparison between the 4 models was significant (*P* < .001) for both the 72-h and 9-day return outcomes. AUC: area under the curve.

LR models using administrative data yielded test AUCs of 0.69 (95% CI 0.68–0.70) and 0.71 (95% CI 0.70–0.72), while XGBoost models using administrative data yielded test AUCs of 0.73 (95% CI 0.72–0.74) and 0.74 (95% CI 0.73–0.74) for 72-h and 9-day return, respectively. XGBoost models using variables available at triage yielded test AUCs of 0.75 (95% CI 0.74–0.76) and 0.75 (95% CI 0.74–0.75), while those using the full set of variables yielded test AUCs of 0.76 (95% CI 0.75–0.77) and 0.75 (95% CI 0.75–0.76) for 72-h and 9-day return, respectively (Figure 2). The training and validation AUC values for each model are provided in Supplementary Tables S2 and S3.

### Variables of importance

**Figure 3.**
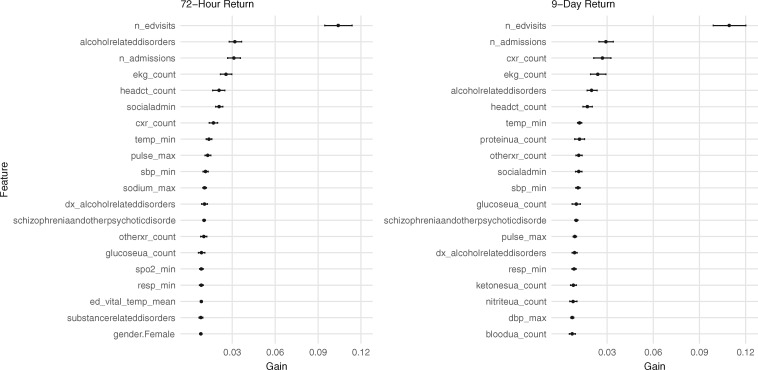
Variables with high information gain for XGBoost models using the full dataset. The top 20 informative variables are shown for each outcome. The points represent the mean information gain from a 100 runs of XGBoost, while the horizontal lines show bootstrapped 95% confidence intervals. Note that information gain does not specify directionality, but instead encodes the predictive value of a variable in a nonlinear model. Description of each variable can be found in Supplementary Table S1.

Variables with high information gain for the full XGBoost models included markers of ED usage such as number of prior ED visits, number of prior admissions, ECG counts, urinalysis counts, as well as historical vital signs and a comorbidity of alcohol- or substance-related disorders (Figure 3). Variables from the current visit, such as the mean temperature during the patient’s current visit, were more informative for predicting 72-h return compared to 9-day return. Reduced XGBoost models using the top 20 variables yielded test AUCs of 0.73 (95% CI 0.71–0.74) and 0.73 (95% CI 0.72–0.74) for 72-h and 9-day return, respectively. Information gain values for the top 100 variables are provided in Supplementary Table S4.

### Statistical measures

Statistical measures for XGBoost models built on data available at triage, at 3 different cutoffs (0.3, 0.5, and 0.7), are shown in Table 3. The cutoff of a prediction model represents the threshold above which a probability output of the model is classified as a positive case and is responsible for the tradeoff between sensitivity and specificity. At a cutoff of 0.5, the models were equivalent to a diagnostic test for 72-h ED return with a sensitivity of 0.16, specificity of 0.99, positive predictive value of 0.75, and a negative predictive value of 0.92 and a diagnostic test for 9-day ED return with a sensitivity of 0.23, specificity of 0.98, positive predictive value of 0.70, and a negative predictive value of 0.87.

**Table 3.** Statistical measures for ED returns model using data available at triage

Outcome	Cutoff	Sensitivity	Specificity	PPV	NPV
72-h return	0.3	0.24	0.98	0.55	0.93
	0.5	0.16	0.99	0.75	0.92
	0.7	0.09	0.99	0.89	0.92
9-day return	0.3	0.35	0.94	0.53	0.88
	0.5	0.23	0.98	0.70	0.87
	0.7	0.15	0.99	0.83	0.86

*Note:* The prevalence of outcome in the test set was 8.9% for 72-h return and 15.9% for 9-day return. Bootstrap 95% CI for all measures <±0.02.

*Abbreviations:* CI: confidence interval; ED: emergency department; NPV: negative predictive value; PPV: positive predictive value.

## DISCUSSION

In this study, we predict 72-h and 9-day ED return using gradient boosting on an expansive set of 330 631 ED discharges and 1500 variables. We are able to predict 72-h and 9-day return with similar accuracy, with small but significant improvement from including comprehensive elements of clinical data. Our best-performing models for predicting 72-h and 9-day ED return yielded test AUCs of 0.75 and 0.76, respectively, while our reduced models built on the top 20 informative variables yielded a test AUC of 0.73 for both outcomes. We expand on a prior model that predicted 72-h return in a Veteran’s Affairs population, enhancing performance by using a nonlinear algorithm on a large number of clinical variables.^12^ Furthermore, we are the first to report measures of diagnostic accuracy for the prediction of 9-day return.^15^

We confirm that visits that result in early ED return differ in several ways from those that do not result in return. Previously identified risk factors such as arrival mode, insurance status, number of previous ED visits, number of hospital admissions, and types of diagnoses were found to be significant.^10–12^^,^^18^^,^^25^ Discharge diagnoses such as alcohol-related disorders, substance-related disorders, and skin infection were over-represented in those visits resulting in early return. Exploratory analysis was not sufficient to reveal any clear differences between visits that result in 72-h return and those that result in 9-day return. However, variables relating to patient’s acuity such as current vital signs were more informative for predicting 72-h return and suggest that 72-h returns may represent a more acute patient population compared to 9-day returns. Given that our study was not designed to delineate differences between these 2 return populations, further studies are needed to support using 9 days as an alternative cutoff to 72 h for early ED return.

The AUCs of our best models do not exceed 0.8 despite the inclusion of numerous clinical variables. The challenge of predicting early ED return may in part be due to the multifactorial nature of ED returns. Although the EHR can accurately encode quantitative variables such as presence of comorbidities and frequency of hospital utilization, a patient’s decision to return to the ED may also be contingent on emotional factors like fear and uncertainty that are not well-captured in the EHR.^16^^,^^42^ Recent studies suggest free-text data such as physician charts may contain information regarding these subjective factors.^43^^,^^44^ Quantitative measurements of psychosocial factors using standardized surveys represent another way to encode this information, although one study’s quantification of uncertainty, the “U-Scale,” was shown to not be predictive of 30-day ED return.^42^^,^^45^ Our reduced models, built on the top 20 informative variables for each outcome, point to the importance of resource over-utilization and history of alcohol or substance use disorder in predicting ED return and may facilitate external replication of our results in different practice settings and patient populations.

We anticipate multiple clinical applications of ED return risk prediction models. It may be valuable to predict ED return visits early in the ED course in order to trigger intervention. We show that most informative variables are available by the time of triage, suggesting that care coordinators or social workers may begin interventions as early as triage. This suggestion is further supported by prior studies that have shown that patient disposition (ie, admission or discharge) can be robustly predicted at triage.^30^^,^^46^ Although conceptual models have shown that social work services may yield net economic benefits, there have been no formal cost-benefit analyses regarding interventions aimed at modulating return visits.^47–49^ Given this fact, we explored various model cutoffs, each which would capture a different number of likely returns with varying positive predictive values. At a cutoff of 0.5, the model built on data available at triage represents a diagnostic test for 72-h ED return with a sensitivity of 0.16, specificity of 0.99, positive predictive value of 0.75, and a negative predictive value of 0.92. Such a model could be used as a prospective rule-in trigger to start intervention at the completion of triage even before the patient is roomed. Well-resourced EDs may choose to use a lower cutoff to try capture more early returns, or to identify different types of returning patient populations.^50–52^ Future efforts will be required to fully understand various subgroups within early returns and to identify which of them will respond to intervention.^2^^,^^13^^,^^53^

Another potential clinical application may be to use the model to retrospectively screen return cases for those associated with lapses in care.^1^ It is increasingly understood that many ED returns do not represent adverse events or lapses in care.^2^^,^^53^ In accordance with prior research, we found that the risk of admission is lower for return visits (22% for 72 h, 20% for 9 days), compared to the general admission risk (30%).^2^ It has been proposed that “expectedness” of patient returns is a key dimension for classifying lapses in care.^15^ While expectedness is currently a subjective assessment, we hypothesize that it may be definable in a data-driven manner as the probability output of a model. In this framing, returns with low probability outputs might be passed on for quality assurance review. Further studies will be needed to assess the validity of this approach.

This study has several limitations. The study is a retrospective study that lacks a separate validation cohort. The study did not take account into scheduled versus unscheduled ED return, given that there is no formal definition for scheduled visits and that scheduling a return visit does not necessarily result in return. The study also restricts patient data to those collected from prior ED visits and does not include data from outpatient clinic or inpatient wards, which may provide important information regarding a patient’s healthcare usage pattern. Although our study included 2 neighboring EDs within the same city, it did not take account of return visits to EDs outside the city or to EDs in a different hospital system and likely under-represents ED return.^9^ Finally, this study does not provide set guidelines on how the information obtained about risk of early ED return may be used to improve patient care.

## CONCLUSION

Machine learning is able to predict 72-h and 9-day return with modest accuracy, with small but significant improvement from the use of clinical variables. The highest impact features are available by the completion of triage, supporting earlier intervention for patients with high risk of return. Further studies are needed for clinical implementation of such a model.

## SUPPLEMENTARY MATERIAL

Supplementary material is available at *Journal of the American Medical Informatics Association* online.

## CONTRIBUTORS

WSH, ADH, and RAT conceptualized the study. WSH and RAT curated the data. WSH preprocessed the data, trained the models, and created the figures. WSH and ADH developed the methodology and wrote the original draft. All authors revised the manuscript and approved its final version. RAT supervised all steps of the work.

## FUNDING

Woo Suk Hong was supported by the Yale School of Medicine Medical Student Research Fellowship. Adrian Daniel Haimovich and Richard Andrew Taylor received no specific funding for this study.

*Conflict of interest statement.* None declared.

## Supplementary Material

ooz019_Supplementary_Data
