# Supplementary material for: Predicting 72-hour and 9-day return to the emergency department using machine learning
Source: JAMIA Open. 2019 Jul 1;2(3):346–52. doi: 10.1093/jamiaopen/ooz019 (PMC6951979; doi:10.1093/jamiaopen/ooz019)
Supplement: ooz019_Supplementary_Data [file ooz019_supplementary_data.zip › TextS1_TableS2_TableS3.docx]

**Supplementary Text S1 (includes Tables S2 and S3)**

***Predicting 72-Hour and 9-Day Return to the Emergency Department Using Machine Learning***

Woo Suk Hong, BS; Adrian Daniel Haimovich, MD, PhD; Richard Andrew Taylor, MD, MHS

*All processing and analysis scripts can be found at: https://github.com/yaleemmlc/edreturn*

**DATA PROCESSING**

The processing steps for demographics, triage vitals, chief complaint, hospital usage statistic, past medical history, outpatient medications, historical vitals and labs, and historical imaging/ECG counts are outlined in the Methods section of the paper: Hong WS, Haimovich AD, Taylor RA. *Predicting hospital admission at emergency department triage using machine learning.* PLOS ONE. 2018;13(7):e0201016. (doi:10.1371/journal.pone.0201016)

We outline the processing steps for additional variables below.

***ED Vitals***

Vital signs were represented by the minimum, maximum, and the mean value of systolic blood pressure, diastolic blood pressure, pulse, respiratory rate, oxygen saturation, presence of oxygen device (1 for any type, 0 for room air), and temperature recorded during the patient’s current ED stay. Values beyond physiologic limits were replaced with missing values prior to calculation.

***ED Labs***

Given the diversity of labs ordered within the ED, the 150 most frequent labs comprising 94% of all orders were extracted then divided into labs with numeric values and those with categorical values. The cutoff of 150 was chosen to include labs ordered commonly enough to be significant in the management of most patients (e.g., Troponin T, BNP, CK, D-Dimer), even if they were not as frequent as routine labs like CBC, BMP, and urinalysis. Categorical labs, which included urinalysis and culture results, were recoded into binary variables with 1 for any positive value (e.g. positive, trace, +, large) and 0 otherwise. Any growth in blood culture was labeled positive as were urine cultures with > 49,000 colonies/mL.

***ED Medications***

Each medication administered during the visit was recoded by taking the first word of the medication order (eg. ‘OXYCODONE 4 MG TABLET’ >> ‘oxycodone’, ‘ONDANSETRON HCL (PF) 4 MG/2 ML INJECTION’ >> ‘ondansetron’), thus ignoring dosing and route of administration. The 100 most frequently administered medications, comprising 93% of all entries, were kept as unique entries and all others binned to ‘Other’. Each variable encoded the cumulative number of times a medication was ordered.

***ED Orders [excluding labs and medications]***

The 40 most frequent orders, comprising 92% of all orders, were kept as unique entries and all others binned to ‘Other’. Each variable encoded the presence or absence of the order during the visit. Imaging orders were recoded into following categories: electrocardiogram (ECG), chest x-ray, other x-ray, echocardiogram, other ultrasound, head CT, other CT, MRI, and all other imaging.

***ED Discharge Diagnosis***

As we had previously done for past medical history, ICD-9 codes for the visit’s discharge diagnosis were mapped onto 281 clinically meaningful categories using the Agency for Healthcare Research and Quality (AHRQ) Clinical Classification Software (CCS), such that each CCS category became a binary variable.

**MODEL FITTING FOR LOGISTIC REGRESSION**

Given that logistic regression is equivalent to a neural network with a single fully connected layer using the sigmoid function, logistic regression was implemented in keras (<https://keras.rstudio.com/>) using the following fixed architecture:

- architecture = 350 (number of variables after one-hot encoding) ⇒ 1
- optimizer = rmsprop
- learning rate = 0.001
- epoch = 2

All other hyperparameters were left to their default values. The training accuracy and the test AUCs are provided below.

**Table S2. Architecture, Training Accuracy, and Test AUCs for Each LR model**

| **Outcome** | **Dataset** | **Architecture/Hyperparameters** | **Training ACC^a^** | **Test AUC** | **95% CI Lower** | **95% CI Upper** |
| --- | --- | --- | --- | --- | --- | --- |
| 72-Hour Return | Administrative | 350 ⇒ 1, optimizer_rmsprop(lr = 0.001), 2 epochs | 0.917 | 0.692 | 0.681 | 0.703 |
| 9-Day  Return | Administrative | 350 ⇒ 1, optimizer_rmsprop(lr = 0.001), 2 epochs | 0.853 | 0.708 | 0.700 | 0.716 |

^a^ *keras* optimizes accuracy rather than AUC.

**MODEL FITTING FOR XGBOOST**

The hyperparameters for the *xgboost* package (version 0.6-4) and their default values are listed in its online documentation (<http://xgboost.readthedocs.io/en/latest/parameter.html>).
The following hyperparameters were tuned to maximize performance on the validation sets:

- max_depth
- colsample_bylevel

The following hyperparameters were fixed:

- nrounds = 20
- eta = 0.3
- nthread = 5
- eval_metric = 'auc'
- objective = 'binary:logistic'

All other hyperparameters were left to their default values. In case of a tie up to the 3rd decimal place, preference was given to the lower-complexity model. The optimized set of hyperparameters for each XGBoost model, as well as the training and validation AUCs, is provided below.

**Table S3. Optimized Hyperparameters and Training, Validation, and Test AUCs for Each XGBoost Model**

| **Outcome** | **Dataset** | **Hyperparameters** | **Training AUC** | **Val AUC** | **Test AUC** | **95% CI Lower** | **95% CI Upper** |
| --- | --- | --- | --- | --- | --- | --- | --- |
| 72-Hour Return | Administrative | max_depth = 15, colsample_by_level = 0.03 | 0.773 | 0.724 | 0.734 | 0.724 | 0.744 |
|  | Triage | max_depth = 15, colsample_by_level = 0.03 | 0.773 | 0.743 | 0.750 | 0.740 | 0.760 |
|  | Discharge | max_depth = 15, colsample_by_level = 0.03 | 0.789 | 0.758 | 0.761 | 0.751 | 0.771 |
|  | Top 20 | max_depth = 10, colsample_by_level = 0.3 | 0.734 | 0.717 | 0.725 | 0.714 | 0.735 |
| 9-Day Return | Administrative | max_depth = 15, colsample_by_level = 0.03 | 0.774 | 0.736 | 0.737 | 0.729 | 0.744 |
|  | Triage | max_depth = 20, colsample_by_level = 0.03 | 0.784 | 0.749 | 0.745 | 0.737 | 0.753 |
|  | Discharge | max_depth = 20, colsample_by_level = 0.03 | 0.805 | 0.759 | 0.752 | 0.745 | 0.760 |
|  | Top 20 | max_depth = 10, colsample_by_level = 0.3 | 0.738 | 0.727 | 0.727 | 0.719 | 0.735 |
